# Can a Variant of the Implicit Association Test Detect Nonsuicidal Self-Injury in a Clinical Population? A Registered Report

**DOI:** 10.32872/cpe.19871

**Published:** 2026-08-31

**Authors:** Femke Cathelyn, Pieter Van Dessel, Tilia Linthout, Laurence Claes, Jan De Houwer

**Affiliations:** 1 Quality Centre for Diagnostics, Ghent, Belgium; 2 Department of Experimental-Clinical and Health Psychology, Ghent University, Ghent, Belgium; 3 Faculty of Psychology and Educational Sciences, University of Leuven, Leuven, Belgium; 4 Faculty of Medicine and Health Sciences, University of Antwerp, Antwerp, Belgium; Friedrich-Alexander-Universität Erlangen-Nürnberg, Erlangen, Germany; Philipps-University of Marburg, Marburg, Germany

**Keywords:** nonsuicidal self-injury, implicit measures, past behaviour, prediction, clinical population, outpatients

## Abstract

**Background:**

Nonsuicidal self-injury (NSSI) is a severe and prevalent mental health problem. Measures to detect which individuals are at risk of engaging in NSSI would be valuable for clinical practice. However, we still lack strong predictors of future NSSI behaviour, with the most notable exception being prior NSSI behaviour. Yet, the measurement of prior NSSI behaviour with self-report measures can be difficult because individuals may be motivated to conceal this harmful behaviour. To overcome this problem, an implicit measure was developed that assesses automatic responding to statements about prior NSSI behaviour (i.e., the Past Nonsuicidal Self-Injury Implicit Association Test: P-NSSI-IAT). Previous studies tested the predictive utility of this measure in online studies with samples of at-risk participants and produced promising results. The current study examines the predictive utility of the P-NSSI-IAT for NSSI in clinical samples.

**Method:**

Outpatients (*N* = 68) completed the P-NSSI-IAT and reported on past-year and past-month NSSI, as well as perceived likelihood of future NSSI.

**Results:**

Patients with recent NSSI had significantly higher IAT scores than those without a history of NSSI. The P-NSSI-IAT predicted past-year NSSI but did not significantly predict past-month NSSI or perceived future likelihood of NSSI. It did not add predictive value beyond self-report risk factors in multivariate models.

**Conclusion:**

These findings suggest that the P-NSSI-IAT may help detect prior NSSI in clinical contexts but offers limited added value for the prediction of NSSI beyond existing self-report measures.

## Highlights

- The P-NSSI-IAT significantly discriminated between clinical patients with and without recent NSSI behaviour.
- IAT scores predicted past-year NSSI but did not significantly predict past-month NSSI or future self-rated likelihood of NSSI.
- The P-NSSI-IAT did not add incremental predictive value over established self-report risk factors in multivariate models.
- Belief-based implicit measures like the P-NSSI-IAT may aid in detecting sensitive behaviours such as NSSI, though their added value beyond self-report remains limited.

Nonsuicidal self-injury (NSSI) is the direct and deliberate destruction of one’s own body tissue without suicidal intent (Nock, 2010) and is a severe health problem. Besides physical scars and infections, NSSI can result in feelings of guilt and shame (Long, 2018; Rosenrot & Lewis, 2020) and evoke stigmatising behaviour from family, peers (Doyle, 2017; Oldershaw et al., 2008), and even health care professionals (Saunders et al., 2012). While NSSI is typically conceptualised as non-suicidal, prior NSSI behaviour has been shown to be a robust risk factor and predictor of suicidal thoughts and behaviours (Franklin et al., 2017; Griep & MacKinnon, 2022; Kiekens et al., 2018; Turner et al., 2013; Whitlock et al., 2013). NSSI is a highly prevalent problem. Lifetime prevalence rates have been estimated at 4%–6% in general adult populations (Klonsky, 2011; Liu, 2023) and up to 45% in adult clinical populations (Andover & Gibb, 2010). Among community (non-clinical) adolescent samples, meta-analytic findings suggest a lifetime prevalence of 16% (Farkas et al., 2024), with even higher rates reported in clinical or younger samples (e.g., Swannell et al., 2014). Given the severity and prevalence of NSSI, it is important for practitioners to assess which patients are at risk of NSSI behaviour. Assessment methods to detect and predict NSSI could provide a valuable opportunity to prevent NSSI and suicidal behaviour.

Potential predictors and risk factors for NSSI have been investigated for over a decade. Nevertheless, results from a recent meta-analysis on studies longitudinally predicting NSSI revealed that most factors show weak predictive utility, suggesting limited clinical value (Fox et al., 2015). One notable exception was prior NSSI behaviour, which has been identified as the strongest risk factor for future NSSI. Results from individual studies have shown that prior self-harm behaviour outperforms other predictors when longitudinally forecasting self-harm (Janis & Nock, 2008), and have revealed that prior NSSI behaviour is the only significant predictor for NSSI at different follow-up moments (Tuisku et al., 2014). Moreover, results from a recent study using machine learning techniques showed that prior NSSI behaviour was the most important predictor across several time points (Fox et al., 2019). As such, assessing prior NSSI behaviour could be a valuable strategy for predicting future NSSI and related behaviours in clinical contexts.

Importantly, however, merely asking individuals about prior NSSI behaviour in clinical settings could be problematic. Patients might be reluctant to disclose NSSI behaviour given the potential negative consequences, such as feelings of shame, fear of stigmatization, and fear of hospitalization (Long, 2018; MacDonald et al., 2020; Simone & Hamza, 2020). The addition of implicit measures to screening procedures for NSSI risk detection could provide a solution to this problem. Unlike self-report measures, implicit measures are used to assess responses occurring under automaticity conditions (De Houwer et al., 2009; De Houwer & Moors, 2012). For instance, studies have shown that responding on certain implicit measures, such as the Implicit Association Test (IAT; Greenwald et al., 1998) and some of its variants, is far less controllable than responding on self-report measures (e.g., Agosta et al., 2011; Egloff & Schmukle, 2002; Kim, 2003; Stieger et al., 2011). As such, these measures could be less susceptible to deception.

Because of their potential benefits over self-report measures, scholars have developed implicit measures for predicting NSSI and other self-harm behaviours (e.g., Nock et al., 2010; Nock & Banaji, 2007). Notably, most of these measures do not target predictors that have previously been shown to be strong forecasters of NSSI (for an exception, see Gray et al., 2021). For instance, one of the most frequently used implicit measures in this domain, referred to as the Self-Injury Implicit Association Test (SI-IAT; Nock & Banaji, 2007), targets self-identification with NSSI behaviour. In the SI-IAT, participants categorise stimuli regarding the self (e.g., the word “me”) and others (e.g., the word “them”) alongside self-injurious stimuli and non-self-injurious stimuli (e.g., pictures of skin that has (not) been cut) as fast as possible using two keys on the keyboard. When participants perform better on trials where stimuli regarding the self and self-injurious stimuli share the same response key than on trials where stimuli regarding the self and non-self-injurious stimuli share the same response key, it is inferred that these individuals automatically identify themselves with self-injurious behaviour. While several studies have shown that the SI-IAT can discriminate between injury groups and non-injury groups (Glenn et al., 2017; Nock & Banaji, 2007; Powers et al., 2021), evidence regarding its utility in prospectively predicting NSSI behaviour has been mixed. Two studies have demonstrated that the SI-IAT predicts NSSI over time (Cha et al., 2016; Glenn et al., 2016), while other studies have failed to find such effects (Cha et al., 2016; Franklin et al., 2014; Glenn & Klonsky, 2011; Powers et al., 2021).

A promising new avenue for NSSI risk detection in clinical practice may involve the development of measures that (a) target predictors shown to be strong forecasters of NSSI and (b) are less susceptible to deception. An implicit measure was recently developed that assesses beliefs regarding past behaviour, referred to as the past nonsuicidal self-injury IAT (P-NSSI-IAT; Cathelyn et al., 2021). The P-NSSI-IAT follows the same procedure as the SI-IAT, with the exception that its stimuli consist of statements (regarding past NSSI behaviour) rather than single words or pictures. If participants respond faster on trials in which the same response key is used for categorizing statements that are inherently true (e.g., “I’m pressing computer keys”) and statements regarding past NSSI behaviour (e.g., “I have carved my skin on purpose”) than on trials where the same response key is used for categorising statements that are inherently false (e.g., “I’m climbing a mountain”) and statements regarding past NSSI behaviour, this might indicate that these individuals automatically endorse the belief that they have engaged in NSSI in the past. Two previous studies investigated the predictive utility of the P-NSSI-IAT and found that this measure discriminated well between participants who reported to have previously engaged in NSSI behaviour (i.e., cutting or carving of the skin) and participants who reported to have never engaged in NSSI. P-NSSI-IAT scores also prospectively predicted NSSI behaviour over one month (Franklin et al., 2017) and predicted this outcome above and beyond other known risk factors for NSSI (i.e., hopelessness, frequency of past NSSI, and prior suicidal thoughts).

Importantly, however, these studies were conducted in general online samples which were recruited through Prolific Academic (https://www.prolific.co/). To examine the clinical utility of the P-NSSI-IAT, it is important to investigate whether these previous findings generalise to a clinical population. In the current study, we will target outpatients and investigate (a) whether and (b) how well the P-NSSI-IAT discriminates between patients who report having recently engaged in NSSI behaviour (i.e., cutting or carving of the skin in the past year and past month) and patients who report having never engaged in NSSI behaviour. We will also examine (c) whether P-NSSI-IAT scores independently predict self-rated past year, past month, and future likelihood of NSSI, and (d) whether P-NSSI-IAT scores predict these outcomes above and beyond known risk factors for NSSI (i.e., gender, age, hopelessness, psychiatric disorders typically related to NSSI, and prior suicidal thoughts; e.g., Fox et al., 2015; Klonsky & Muehlenkamp, 2007). Note that the main aim of this study is to validate the P-NSSI-IAT by assessing its ability to detect prior NSSI behaviour in a sample of clinical patients. For practical reasons, we do not assess the predictive validity of the P-NSSI-IAT. We include a future likelihood measure for exploratory purposes (see Cathelyn et al., 2021). Appendix A provides an overview of the research questions, hypotheses, sampling plan, analysis plan, and interpretations given different outcomes.

## Method

### Participants

We targeted patients who receive outpatient treatment for various conditions. Participants were recruited through clinical psychologists in Flanders. Recruitment materials explicitly invited individuals with and without a history of NSSI to participate, to allow for comparison across groups. We contacted clinicians through the Flemish Association for Clinical Psychologists, Facebook groups for licensed clinical psychologists, mental health outreach events, and a website where Flemish people can search for licensed clinical psychologists. Clinicians willing to collaborate were asked to invite patients to participate in the study if (a) their first language is Dutch, (b) they were capable of conducting the study (i.e., not having a cognitive impairment and not being in serious crisis), and (c) they were between 18 and 30 years old. This latter inclusion criterion was applied because NSSI behaviour is more prevalent in this age group than in older age groups (Swannell et al., 2014). The patients’ eligibility to participate in the study was evaluated by the clinicians. Participants were reimbursed for their time and effort with a voucher (€10).

A total of 207 participants started the study. Of those, 28 participants dropped out after reading the study information. The data of participants were excluded if they did not provide complete (questionnaire and/or P-NSSI-IAT) data (32 participants). As recommended by Greenwald et al. (2003), we also excluded the data of participants with response latencies below 300 ms on 10% or more of critical P-NSSI-IAT trials or with P-NSSI-IAT error rates above 30% across the entire task, and/or above 40% for any of the critical blocks (34 participants). We applied these exclusion criteria for several reasons. First, the researchers had minimal control over what participants were doing as the study was conducted online. Second, unlike student participants who completed most past IAT studies, the participants in the current study were likely not familiar with reaction time measures. As such, more participants might have failed to adhere to instructions than in previous IAT studies. Third, the current sample is relatively small; thus, the impact of outlier scores may be stronger. Finally, participants might have been motivated to conceal their NSSI behaviour and thus might have tried to alter their P-NSSI-IAT scores. Studies show that participants who try to alter their IAT scores tend to produce higher error rates (e.g., because they believe that increasing the number of errors is a valid faking strategy, Röhner et al., 2013; Steffens, 2004). However, for exploratory purposes, we also conducted the analyses including the data of participants who met the latter exclusion criteria and will report whether doing so affected the results.

Further, we excluded the data of participants who could not be assigned to the past year NSSI group or to the no history of NSSI group (45 participants). This occurred when participants indicated they had not engaged in skin cutting in the past year, but had (a) engaged in skin cutting more than a year ago, (b) used another NSSI method during their lifetime, and/or (c) attempted suicide using cutting of the skin as a method (because the category labels and items of the P-NSSI-IAT do not distinguish between suicidal and non-suicidal self-injury). Relatedly, we had planned to exclude participants if there was uncertainty regarding their NSSI group status (e.g., because of inconsistencies in the self-report data, for example, when participants report having engaged in NSSI in the past month, but not in the past year), but no participants met this criterion.

The main effect of interest is the difference in P-NSSI-IAT scores between the past NSSI group and the no history of NSSI group. In line with previous studies (Cathelyn et al., 2021), we tested differences in P-NSSI-IAT scores between individuals who had never engaged in NSSI on the one hand and individuals who had engaged in NSSI on the other hand. In the latter group, we distinguished between individuals who had engaged in NSSI in the past year and individuals who had engaged in NSSI in the past month. Given that it is more feasible to recruit a sufficient number of participants who have engaged in NSSI in the past year than a sufficient number of participants who have engaged in NSSI in the past month, the required sample sizes for the current study were calculated based on the estimated effect size for an independent samples *t*-test comparing P-NSSI-IAT scores from the past year NSSI group and the no history of NSSI group. Note that previous studies testing the predictive utility of implicit measures for NSSI typically focused on detecting lifetime prevalence of NSSI, but this may be less relevant for clinical purposes (Powers et al., 2021). We focus on more recent behaviour and calculate the power of the study to detect past year NSSI which is of clinical importance. According to conventions for effect sizes (Cohen, 1988), we found medium to large effect sizes in our previous studies when comparing P-NSSI-IAT scores between a past year NSSI group and a no history of NSSI group (with *d*s ranging from 0.68 to 0.82; Cathelyn et al., 2021). Given that previous studies have demonstrated that group differences between prediction scores for NSSI tend to be more modest in clinical than in community samples (Franklin et al., 2017; Sohn et al., 2021), we applied an estimated effect of *d* = .60 in the power analysis, which is slightly smaller than the lowest observed effect size.

Results of the power analysis showed that a total of 146 participants would allow for detecting the estimated effect size with 85% power in a one-tailed *t*-test (α = .05) with an estimated group-allocation ratio (i.e., the proportion of cases and controls) equal to 5 (i.e., 24 cases and 122 controls). Because our previous studies were conducted in general online samples, and we conducted pre-screening studies to recruit many cases, we could not base the estimated group-allocation ratio on our previous studies. Therefore, we chose to apply a conservative estimate of the group allocation ratio in the power analysis and registered that we would stop data collection when 146 participants fully completed the study.

While we indicated in our preregistered protocol that data collection would stop when 146 participants had fully completed the study, we noted during data collection that our power analysis was based on retaining 146 participants after applying predefined exclusion criteria (e.g., based on IAT performance or unclear group classification). Due to substantial and prolonged difficulties in recruiting participants from the targeted clinical population, high exclusion rates, and given that participants were compensated upon completion of the study procedure regardless of later data exclusion, we ceased data collection due to time and financial constraints after three full years of recruitment. At that point, 147 participants had completed the full study and received reimbursement.

After applying the preregistered exclusion criteria, the final analysed sample consisted of 68 participants (mean age = 30, *SD* = 12; 24% male, 75% female, 1% other identity), including 45 participants who engaged in NSSI in the past year and 23 participants without a history of NSSI. A sensitivity power analysis (G*Power 3.1) indicated that this final sample size provides us with 75% power to detect the estimated effect size of *d* = 0.60. Participants from the past NSSI group reported engaging in skin cutting 4.31 on average over the past year (*SD* = 3.17). Based on self-report symptom screening, 44% of participants reported symptoms consistent with a mood disorder, 46% with an anxiety disorder, 21% with an eating disorder, and 25% with a substance use disorder. Details on screening criteria are provided in the Measures of Risk Factors section.

### Materials and Apparatus

The study was built using lab.js, a tool for creating browser-based studies. The study was hosted online, and participants received a link to the study via their treating clinician and were asked to complete the study using their laptop or desktop. All materials were translated into Dutch using back-translation, except for the Patient Health Questionnaire (PHQ; Spitzer et al., 1999). We used the Dutch version of the PHQ as translated by the MAPI Research Institute (see https://www.phqscreeners.com/).

#### P-NSSI-IAT

The P-NSSI-IAT followed the same procedure as in previous studies (Cathelyn et al., 2021). Participants were instructed to categorise statements regarding past (non-) NSSI behaviour and statements that are inherently true or false as fast as possible using the E- or I-key on the keyboard. On each trial, a statement appeared in the middle of the screen until participants pressed one of the valid response keys. If the response was correct, the statement disappeared, and the next statement was presented 400 ms later. If the response was incorrect, a red cross replaced the statement for 200 ms, and the next statement appeared 400 ms after the red cross appeared. Category labels were presented in the top left and right corners of the screen to aid categorization. The category labels and stimuli are listed in Appendix B. We used the font Helvetica with font size 15 for the category labels and font size 18 for the statements. The category labels were presented in upper case letters, and the statements were presented in lower case letters. The P-NSSI-IAT procedure was presented full screen.

The P-NSSI-IAT consists of seven blocks and 192 trials. Before the first block began, participants received instructions emphasizing speed and accuracy. Specifically, they were asked to respond as quickly as possible while trying to avoid too many mistakes, to avoid distractions, to pay attention, and to keep their fingers on the E and I keys throughout the task. The full task instructions are provided in Appendix B. Before the start of all subsequent blocks, participants were reminded to try to respond as quickly as possible. Participants were able to proceed to the next block by pressing the space bar.

In the first block of the P-NSSI-IAT, participants practiced categorizing four statements regarding past NSSI behaviour (e.g., “I have carved my skin on purpose”) using the E-key, and four statements regarding past non-NSSI behaviour (e.g., “I have carved my skin not once”) using the I-key. In the second block, participants practiced categorizing four statements that are inherently true (e.g., “I’m pressing computer keys”) using the E-key, and four inherently false statements (e.g., “I’m playing football”) using the I-key. Both practice blocks consisted of 32 trials during which the eight statements were each presented four times. In the next two blocks, participants categorised statements from all four categories simultaneously using the practiced key assignment (i.e., the E-key for sentences regarding past NSSI behaviour and statements that are inherently true, and the I-key for sentences regarding past non-NSSI behaviour and sentences that are inherently false). In the combined blocks (16 and 32 trials) the 16 statements were each presented three times. Following this, there was another practice block in which only statements regarding past (non-) NSSI behaviour needed to be categorised, but this time with the response key assignment reversed (i.e., the E-key for statements regarding past non-NSSI behaviour and the I-key for statements regarding past NSSI behaviour). This practice block consisted of 32 trials during which the eight statements were each presented four times. In the final two combined blocks, participants categorised statements from all four categories using the new response key assignment. The order of the trials was determined randomly for each block and participant.

#### Self-Report Measures of NSSI Behaviour

To assess the outcome variables of interest, participants were asked how many times they had intentionally cut or carved their skin without suicidal intent in the past 12 months and in the past 30 days. These two questions were rated on a scale ranging from 0 to 10+ times. Participants were also invited to indicate how likely they would be to intentionally cut or carve their skin without intending to kill themselves in the future. This question was rated on a Likert scale ranging from 0 (*low/little*) to 4 (*very much/severe*).

To determine whether the data of participants should be excluded (see exclusion criteria), participants were also asked about lifetime skin cutting with and without suicidal intent, and lifetime NSSI using any other method. Participants were asked to indicate “yes” or “no” when answering these questions. All questions regarding NSSI behaviour were based on or adapted from the Deliberate Self-Harm Inventory (DSHI; Gratz, 2001) and the Self-Injurious Thoughts and Behavior Interview (SITBI; Nock et al., 2007).

#### Measures of Risk Factors

Indicators of psychiatric diagnoses were assessed using the PHQ, a self-administered questionnaire that screens for five common types of psychiatric disorders: mood, anxiety, eating, substance abuse, and somatoform disorders. Note that we only included the first four modules of the questionnaire because these disorders are frequently related to NSSI (e.g., Klonsky & Muehlenkamp, 2007). Hopelessness was assessed using the Beck Hopelessness Scale (BHS; Beck et al., 1974). The BHS assesses positive and negative beliefs about the future and consists of 20 items (e.g., “my future seems dark to me”). Participants were asked to evaluate these statements as true or false. Finally, the frequency of prior suicidal thoughts was assessed through a question that was adapted from the SITBI: “During how many separate times in your life have you had thoughts of killing yourself?”. Participants indicated this frequency on a scale ranging from 0 to 10+ times.

### Procedure

After providing informed consent, participants indicated their age and gender and completed the P-NSSI-IAT. Afterwards, participants were reminded about the anonymous nature of the study (to reduce socially desirable responding) and answered the questions regarding NSSI behaviour. In the next phase, participants completed the PHQ, the BHS, and prior suicidal thoughts. At the end of the study, participants were referred to several help sources for coping with NSSI and suicidal thoughts and behaviour. Participants were also referred to a separate website where they were asked to provide the information needed for compensation purposes.

### Data Pre-Processing

To calculate P-NSSI-IAT scores we used the D4 scoring algorithm (Greenwald et al., 2003). This algorithm assesses the difference in reaction times between the first two and the second two combined blocks while correcting the latencies for individual variability. In line with this algorithm, trials with latencies longer than 10,000 ms were removed and latencies on trials with an incorrect response were replaced with the participant’s block mean (based on correct responses) plus a 600 ms penalty. Reaction times on trials of the first two combined blocks were subtracted from reaction times on trials of the second two combined blocks, such that higher scores indicate faster responding during combined blocks in which statements regarding past NSSI behaviour and statements that are logically true share the same response key. The Spearman-Brown corrected split-half reliability for the P-NSSI-IAT was .88, based on the correlation between D-scores computed from odd- versus even-numbered trials.

The presence of any mood, anxiety, eating or substance abuse disorder indicators was determined using the manual and scoring instructions for the PHQ (Spitzer et al., 1999). No distinction was made between specific disorders (e.g., major depressive disorder, other depressive syndrome, etc.). If a participant met the scoring threshold for any specific disorder subtype (e.g., major depressive disorder), the overarching category (e.g., mood disorder) was scored as “1”, otherwise it was scored as “0”. The Cronbach's alpha for the modules of the PHQ were .90 (mood disorders), .71 (anxiety disorders), .59 (eating disorders), and .71 (substance abuse disorders). For hopelessness, each BHS item indicative of hopelessness was scored as “1”, and total scores were calculated by summing the 20 items (Cronbach’s alpha = .91).

To assign participants to the NSSI groups, we used the self-reported NSSI frequencies and ratings. Table 1 provides an overview of the criteria of the self-report questionnaires that were used to assign participants to the NSSI groups and includes the number of participants per NSSI group.

**Table 1:** Grouping of Participants and Number of Participants per NSSI Group

Group	*n*	NSSI frequencies and ratings		
		Past year NSSI	Past month NSSI	Future likelihood NSSI
Past year NSSI group	45	> 0	= 0 or > 0	–
Past month NSSI group	23	> 0	> 0	–
No history of NSSI group	23	= 0	= 0	–
Low future likelihood NSSI group	26	= 0 or > 0	= 0 or > 0	0 – 2
High future likelihood NSSI group	42	= 0 or > 0	= 0 or > 0	3 – 4

*Note.* NSSI = nonsuicidal self-injury.

## Results

### Group Differences in P-NSSI-IAT Scores

To examine whether P-NSSI-IAT scores distinguish between participants with and without a history of NSSI, we conducted two independent samples *t*-tests. First, participants who reported no lifetime history of NSSI (*M* = 0.04, *SD* = 0.54) had significantly lower IAT scores than participants who reported engaging in past-year NSSI (*M* = 0.42, *SD* = 0.48), *t*(66) = 2.96, *p* = .002, *d* = 0.76. Second, participants without a history of NSSI also had lower IAT scores than participants who reported engaging in past-month NSSI (*M* = 0.34, *SD* = 0.57), *t*(44) = 1.85, *p* = .036, *d* = 0.54.

Receiver operating characteristic (ROC) analyses were used to assess how well P-NSSI-IAT scores discriminated between individuals with and without a history of NSSI. For past-year NSSI, the area under the curve (AUC) was 0.72 (95% CI [0.58, 0.86]), indicating good discriminative ability. In previous studies (Cathelyn et al., 2021), we established cut-off scores for the P-NSSI-IAT to maximise sensitivity (0.16) or specificity (0.65) while retaining fair specificity and sensitivity, respectively. At this sensitivity-maximizing cutoff, sensitivity was 0.78 and specificity was 0.57. At the specificity-maximizing cutoff, sensitivity dropped to 0.36, while specificity increased to 0.87. When using a cutoff of 0.00 (a theoretically relevant cut-off; Cvencek et al., 2021), sensitivity was 0.82, and specificity was 0.43.

For past-month NSSI, the AUC was 0.68 (95% CI [0.52, 0.85]). Sensitivity and specificity at the 0.16 cutoff were 0.74 and 0.57, respectively. At the 0.65 cutoff, sensitivity was 0.39 and specificity was 0.87. Using a cutoff of 0.00 yielded a sensitivity of 0.74 and specificity of 0.43.

### Predictive Validity

We conducted three logistic regression analyses to test whether P-NSSI-IAT scores independently predicted past-year NSSI, past-month NSSI, and self-rated future likelihood of NSSI. P-NSSI-IAT scores significantly predicted past-year NSSI (*OR* = 4.19, 95% CI [1.53, 13.10], *p* = .008), but not past-month NSSI (*OR* = 2.76, 95% CI [0.94, 9.37], *p* = .078), or self-rated likelihood of future NSSI (*OR* = 2.75, 95% CI [0.87, 10.75], *p* = .108).

To evaluate the incremental predictive value of the P-NSSI-IAT above and beyond self-report risk factors, we conducted hierarchical logistic regression analyses. Variance inflation factors (VIFs) for all predictors were below 2 (range: 1.06–1.95), indicating no problematic multicollinearity. For past-year NSSI, risk factors (hopelessness, suicidal thoughts, and mood and anxiety disorder indicators) entered in Step 1 significantly predicted NSSI, χ^2^(4) = 40.28, *p* < .001. Suicidal thoughts (*OR* = 1.80, 95% CI [1.29, 2.91]) and mood disorder indicators (*OR* = 8.52, 95% CI [0.97, 117.57]) showed the strongest associations. Adding the P-NSSI-IAT in Step 2 improved model fit, but this improvement was not statistically significant, Δχ^2^(1) = 2.51, *p* = .113.

Similar results were found for past-month NSSI: Step 1 model χ^2^(4) = 43.53, *p* < .001; Δχ^2^(1) for Step 2 = 0.05, *p* = .827; and for self-rated likelihood of future NSSI: Step 1 model χ^2^(3) = 28.09, *p* < .001; Δχ^2^(1) for Step 2 = 0.32, *p* = .570. For past-month NSSI, suicidal thoughts (*OR* = 2.19, 95% CI [1.34, 5.10]) and mood disorder indicators (*OR* = 50.43, 95% CI [3.25, 2471.61]) were associated with higher odds. For future likelihood, hopelessness was the only significant predictor (*OR* = 1.32, 95% CI [1.10, 1.65]).

## General Discussion

This study evaluated the utility of the P-NSSI-IAT as a tool for detecting past NSSI behaviour in a clinical population. Whereas prior research demonstrated the predictive and discriminative potential of this implicit measure in general population samples (Cathelyn et al., 2021), the present findings are the first to extend this work to a sample of outpatients, thereby addressing the clinical relevance of the P-NSSI-IAT.

Our findings support the discriminative validity of the P-NSSI-IAT in this clinical context. Patients who reported past-year or past-month NSSI exhibited significantly higher IAT scores than patients with no history of NSSI, with medium to large effect sizes. ROC analyses further indicated that the P-NSSI-IAT was able to distinguish patients with and without recent NSSI with fair to good accuracy (AUCs of 0.68–0.73). Sensitivity and specificity estimated at pre-established and theory-informed cutoffs were comparable to those observed in prior research, suggesting robustness across samples and potential applicability in clinical screening contexts.

Logistic regression analyses showed that P-NSSI-IAT scores independently predicted past-year NSSI, providing further support for its clinical relevance. However, effects were weaker and not statistically significant for past-month NSSI or perceived future likelihood of NSSI. Moreover, when known risk factors, such as hopelessness, suicidal thoughts, and mood and anxiety disorder indicators, were statistically controlled for, the IAT no longer significantly improved predictive accuracy. These results suggest that while the IAT scores may capture relevant aspects of NSSI-related beliefs, their added value over self-report instruments is currently limited in multivariable prediction models. This might imply that the IAT may mainly be useful in settings where patients are unwilling or unable to disclose NSSI.

A notable pattern in the findings is that the P-NSSI-IAT predicted past-year NSSI, but not past-month NSSI or anticipated future likelihood of NSSI. First, with regard to past-month NSSI, one likely explanation is limited statistical power: the past-month group was the smallest of the three and may not have allowed for the detection of effects of moderate size. We therefore refrain from drawing strong conclusions about the apparent difference between past-year and past-month NSSI. Second, regarding the future likelihood of NSSI, this variable was assessed via a single self-rated expectation, which may be influenced by factors beyond past behaviour, such as motivation to recover, social desirability, limited insight, self-efficacy expectations, or perceived ability to regulate emotions. These factors may contribute to divergence between explicit expectations and the tendencies captured by the P-NSSI-IAT. Future studies should examine whether P-NSSI-IAT scores can meaningfully predict future behaviour when assessed in a longitudinal design.

Several limitations of the study merit consideration. First, the sample size, though adequate for detecting large effects, limited power for identifying smaller effects, particularly for the recent NSSI group. This may partly explain why some effects were in the expected direction but did not reach statistical significance. In addition, group sizes were unequal in several key comparisons (e.g., *N* = 45 for the past-year NSSI group vs. *N* = 23 for the no-history group), which may reduce the precision of some estimates and limit confidence in the stability of the findings. Because unequal group sizes can increase the likelihood of sampling error and affect the reliability of statistical estimates, particularly in small samples, future studies with larger and more balanced groups are needed to replicate these results.

Second, although a clinical sample was recruited, the online design prevented confirmation of diagnoses and limited our ability to characterize heterogeneity in treatment status or context. This restricts conclusions about whether the IAT performs differently across clinical subgroups. Future in-person studies with structured assessments (e.g., structured interviews) would allow more fine-grained examination of such differences. Third, the use of self-report measures for key variables, including NSSI history, recent episodes, and risk factors, may have introduced underreporting or misclassification, given the socially sensitive nature of NSSI. Such inaccuracies could weaken observed group differences, reduce the predictive strength of odds ratios, and lower the apparent discriminative accuracy of the P-NSSI-IAT. Future research may benefit from incorporating clinician assessments or additional corroborating information (e.g., clinical records or ecological momentary reporting). Finally, although self-rated future likelihood of NSSI was included as an exploratory outcome, the absence of longitudinal follow-up means that the current study cannot draw conclusions about actual prospective predictive validity. Longitudinal designs will be essential to evaluate whether the P-NSSI-IAT can meaningfully predict future NSSI behaviour.

Taken together, these findings provide initial evidence that the P-NSSI-IAT can be a valid tool for detecting past NSSI in clinical populations. Although the P-NSSI-IAT may prove useful in contexts where disclosure is limited, it is important to note that we found no evidence for incremental predictive value above self-report risk factors in this clinical sample. Future research could evaluate the IAT’s utility in prospective designs, examine cutoffs tailored to specific clinical contexts, and test whether combining implicit and explicit indicators enhances decision-making in clinical assessments of NSSI.

## Supplementary Materials

The Supplementary Materials contain the following items:

- **Preregistration for the study** (Cathelyn et al., 2023S)
- **Online appendices** (Cathelyn et al., 2026S):
  - *Appendix A.* Overview of the preregistered research questions, hypotheses, sampling plan, analysis plan, and interpretation of possible outcomes.
  - *Appendix B.* Full Past Nonsuicidal Self-Injury Implicit Association Test (P-NSSI-IAT) materials, including category labels, stimuli, and task instructions.
- **Study materials, processing and analysis code, (pseudonymised) raw and processed data** (Linthout et al., 2026S)

CathelynF.
LinthoutT.
Van DesselP.
ClaesL.
De HouwerJ.
 (2023S). Can a variant of the Implicit Association Test detect nonsuicidal self-injury in a clinical population? A registered report
[Preregistration]. PsychOpen. 10.23668/psycharchives.12576


CathelynF.
Van DesselP.
LinthoutT.
ClaesL.
De HouwerJ.
 (2026S). Supplementary materials to "Can a variant of the Implicit Association Test detect nonsuicidal self-injury in a clinical population? A registered report"
[Online appendices]. PsychOpen. 10.23668/psycharchives.22340


LinthoutT.
Van DesselP.
CallewaertL.
 (2026S). Can a variant of the Implicit Association Test detect nonsuicidal self-injury in a clinical population? A registered report
[Research data, code, and materials]. PsychOpen. https://osf.io/qb6d8


## Data Availability

All materials, processing and analysis code, (pseudonymised) raw and processed data are available on the Open Science Framework (https://osf.io/qb6d8).

## Preregistration

The accepted protocol is registered at PsychArchives: https://doi.org/10.23668/psycharchives.12576

## Reporting Guidelines

This study is reported in accordance with the APA Journal Article Reporting Standards (JARS).
